# The Many Faces of Trichotillomania

**DOI:** 10.4103/0974-7753.66916

**Published:** 2010

**Authors:** Preeti Parakh, Mona Srivastava

**Affiliations:** Department of Psychiatry, Institute of Medical Sciences, Banaras Hindu University, Varanasi, India

**Keywords:** Trichotillomania, hair-pulling, obsessive-compulsive spectrum disorders, hair-loss

## Abstract

Trichotillomania is a poorly understood complex disorder of multifaceted pathology which often requires an interdisciplinary approach for management. While sharing similarities with obsessive-compulsive disorder, compelling differences between these have also been noted. We present three cases of trichotillomania highlighting the remarkable variability seen in the presentation of this disorder. In doing so, we discuss on resolving the classificatory issues and call for a consensus on the understanding of the disorder.

## INTRODUCTION

Trichotillomania is characterized by recurrent and persistent urges to pull out one’s own body hair. It occurs *de-novo*, is tension relieving, and cannot be explained by a medical or psychiatric morbidity.[1] The term was first coined in 1889 by the French dermatologist Hallopeau, who described a young man who pulled out his hair in tufts.[2] The word is derived from the Greek *thrix* (hair), *tillein* (to pull), and *mania* (madness).[3] The scalp is reported as the most common site affected, followed by eyelashes, eyebrows, the pubic hair, body hair, and facial hair.[3] This self-inflicted hair-loss can range from small, barely noticeable patches on various areas of the body to total baldness.[3]

The current understanding of chronic uncontrolled hair-pulling as a syndrome is that of an impulse control disorder.[1] However, hair-pulling can also be a symptom with numerous etiologies.[3] The symptom may not be labeled as a syndrome but it occurs outside a person’s awareness, causes significant morbidity, and needs to be addressed for optimal care.[3,4] The current proposals for a revamping of the existing classificatory system is needed to emphasize the existing confusion regarding hair-pulling.[5] Researchers are of the opinion that the term ‘trichotillomania’ be replaced by hair-pulling and that it should include various pathologies.[6] The most accepted view is to place the disorder under anxiety disorders’ category.[5,6]

To highlight the varied presentation of hair-pulling as a symptom associated with significant serious co-morbidities, we are presenting a series of three cases. It is important to resolve the confusion associated with hair-pulling symptom versus hair-pulling syndrome, since it is directly related to appropriate patient care, as these patients can present to a psychiatrist and a dermatologist, more so to the latter.[3] The cases we are going to discuss below, had all first been seen by dermatologists, diagnosed with trichotillomania, and then referred to us for psychiatric consultation. The cases had certain unique features which shall be discussed.

## CASE REPORTS

### Case 1

A 4-year-old boy was referred by a dermatology clinic with complaints of hair-pulling from the right side of the scalp for the last 2 years. A comprehensive psychiatric assessment was done and it revealed a history of episodic abnormal movements of limbs at 1 year 6 months of age, which continued for about 6 months and were followed by the onset of hair-pulling. There was no history of any birth complications or developmental delay. There was no family history of any psychiatric illness or of seizure disorder. An electro-encephalographic examination was done and was suggestive of generalized seizure disorder. No abnormality was detected on the computerized tomography of the brain. The patient was put on valproate (20 mg/kg body weight). A significant decrease in hair-pulling was reported by the parents at the next follow-up. Within 6 months, the bald patch had visibly reduced in size and the hair-pulling had become only occasional. The patient did not fulfil the criteria for trichotillomania.[1]

### Case 2

A 16-year-old boy was referred by the dermatology clinic with a diagnosis of trichotillomania. He had been pulling out his hair for the last 5 years. On examination, there were multiple small bald patches on the scalp. He also had bluish discoloration of the tongue, clubbing of nails, a systolic murmur, hearing impairment, and morphologically and behaviorally appeared to have subnormal intelligence. He was found to have a history of frequent spells of breathlessness, for which no medical consultation had been sought so far. There was no history of any birth complications or developmental delay. There was no family history of any psychiatric illness. A cardiology consultation was requested and an echocardiography done. The patient was found to have Tetralogy of Fallot and was advised surgery. He was referred to a higher centre for cardiothoracic surgery. His IQ was assessed using Seguin form board test and Vineland Social Maturity Scale (VSMS), and he was found to have mild mental retardation (World Health Organization [WHO]). The patient did not meet the specified criteria for trichotillomania.[1] There was marked improvement in hair-pulling on haloperidol in the dose of 2.5 mg/day.

### Case 3

A 71-year-old female was referred by the dermatology clinic with complaints of hair-pulling from the scalp for the last 10 days. She was undergoing treatment for Parkinson’s disease since the last year and was maintaining well. No significant changes had been made in her treatment since the last month. There was a past history of right-sided hemiparesis, 3 years back, from which she had recovered completely. There was no past and family history of any psychiatric illness. The patient was kept under observation and counseled regarding behavioral treatment, on which she reported spontaneous resolution of the hair-pulling over the next 6 weeks. This patient also did not fulfil the criteria of trichotillomania.[1]

## DISCUSSION

All the three cases presented by us did not meet the diagnosis of trichotillomania.[1] However, each one of them had sought consultation for hair-pulling. The etiology of hair-pulling was different in all the cases, viz., in the first case it was a manifestation of seizures, in the second it was probably due to dyspnoeic distress induced anxiety or it could have been a tic disorder as tic is often associated with mental subnormality.[4] In the third case, it was probably iatrogenic because dopamine agonists given as treatment for Parkinsonism are known to precipitate tics. The etiologies forwarded are probable, since the exact pathophysiology in each of the cases is unknown as none of the cases could meet the criteria of trichotillomania.[1] Henceforth we shall be using the term ‘trichotillomania’ in the text in reference to the symptom of trichotillomania.[3,4]

Although the etiology of trichotillomania remains unknown, theories regarding the possible causative factors have evolved considerably over the last decade.[7] Current hypotheses suggest that trichotillomania may be a part of a spectrum of obsessive-compulsive disorders (OCDs), which is a group of disorders sharing features similar to those of OCD and characterized by repetitive disturbing thoughts and/or repetitive stereotyped behaviors; however, there are exceptions to this feature. The relief in symptoms of trichotillomania by the use of various psychotropic agents (including selective serotonin-reuptake inhibitors) or behavior therapy, which are also of use in OCDs, further supports this hypothesis.[6,7]

However, an examination of the literature presents a mixed picture in this regard (trichotillomania), and this aspect has particularly led to a need for revision of the current state of our understanding.[5,6] On one hand, there is a documented link between OCD and trichotillomania,[8] while on the other there is a clear consensus to consider trichotillomania as an anxiety disorder[6] Some neurobiological differences have been detected between OCD and trichotillomania.[9] When subjects with OCD were challenged with the serotonin partial agonist meta-chlorophenylpiperazine (*m*-CPP), they demonstrated blunted neuroendocrine responses, while those with trichotillomania showed no significant differences when compared with normal controls.[9] Magnetic resonance imaging has also detected morphometric differences between OCD and trichotillomania;[10] hence, it is hypothesized that OCD is a result of dysfunction within the frontocaudate pathway and trichotillomania because of the sensorimotor-putamen pathway.[10]

Psychiatric comorbidity is known to be frequent in trichotillomania[3] like major depression, generalized anxiety disorder, OCD, other anxiety disorders, eating disorders, and substance abuse.[3,4] None of the cases that we reported had any psychiatric comorbidity. Instead, all of these cases had some significant medical co-morbidity, which was not in any way related to OCD or OCD spectrum disorders. None had any family history of psychiatric disorders. The first case had a very young age of onset which is a rare finding.[3,7] Resolution of trichotillomania on treatment of the comorbid medical illness shows the association between the two conditions in the particular case.

The variable presentation and complex etiologies of trichotillomania in the above cases is representative of the confusing picture and stance of the diagnostic systems.[4] This calls for a revision of the current understanding of the phenomenon and clarity regarding the symptom versus the syndrome. Although trichotillomania appears to affect a significant number of individuals, it presents in multiple ways in the clinical setting and, in most aspects, remains a poorly understood entity, with some of the most basic questions concerning etiology and management unanswered.[7] Further research is needed to resolve the conflicting views regarding the disorder and to ascertain the associated comorbidities to further the patient care and to arrive at a consensus; the fifth edition of Diagnostic and Statistical Manual of Mental Disorders (DSM-V) may perhaps be helpful in this regard.
